# A Spontaneously Ruptured Hepatic Metastasis From a Gastric Gastrointestinal Stromal Tumor That Presented as Hemoperitoneum

**DOI:** 10.1177/2324709613512475

**Published:** 2013-11-25

**Authors:** Jung-Hee Yoon

**Affiliations:** 1 Inje University, Busan, Korea

**Keywords:** liver, hemorrhage, computed tomography, hemoperitoneum, gastrointestinal neoplasms, diagnosis

## Abstract

Spontaneous hepatic hemorrhage is a rare condition that may be caused by an underlying hepatic tumor, most commonly hepatocellular carcinoma or hepatic adenoma. A spontaneous rupture of a hepatic metastasis from a gastric gastrointestinal stromal tumor is also extremely rare, and the majority of affected patients present with hypovolemic shock or an acute abdomen. In this article, we report the case of a 65-year-old man with a spontaneous rupture of a hepatic metastasis from a gastric gastrointestinal stromal tumor that presented as hypovolemic shock. Cross-sectional imaging studies (computed tomography or magnetic resonance imaging) play a significant role in the diagnosis of this condition and guides its management.

## Introduction

Gastrointestinal stromal tumors (GISTs) are relatively common subepithelial tumors that occur most frequently in the stomach, small bowel, esophagus, and omentum. The liver is the most common metastatic site of a GIST. The spontaneous rupture of a hepatic metastasis from a gastric GIST is extremely rare. To the best of our knowledge, only 2 cases have been reported in the English literature to date. The present report describes the case of a patient who presented with hemoperitoneum and hypovolemic shock due to the spontaneous rupture of a liver metastasis from a malignant gastric GIST.

## Case Description

A 65-year-old man presented at the emergency department of our hospital with sudden right upper quadrant and chest pain, respiratory difficulty, and a 3-day history of fever, chills, nausea, and vomiting. During physical examination, his blood pressure was 100/70 mm Hg, and his pulse rate was 78 beats/min. The results of a peripheral blood examination were the following: white blood cells 10 370/mm^3^, red blood cells 12.6 mg/dL, platelet count 113 000/mm^3^, and C-reactive protein 0.06 mg/dL. The liver function test results were the following: aspartate aminotransferase 178 IU/L (normal range = 7-38 IU/L), alanine aminotransferase 201 IU/L (normal range = 4-43 IU/L), alkaline phosphatase 254 IU/L (normal range = 30-115 IU/L), and α-fetoprotein 1.8 ng/mL (within normal limits).

**Figure 1.**
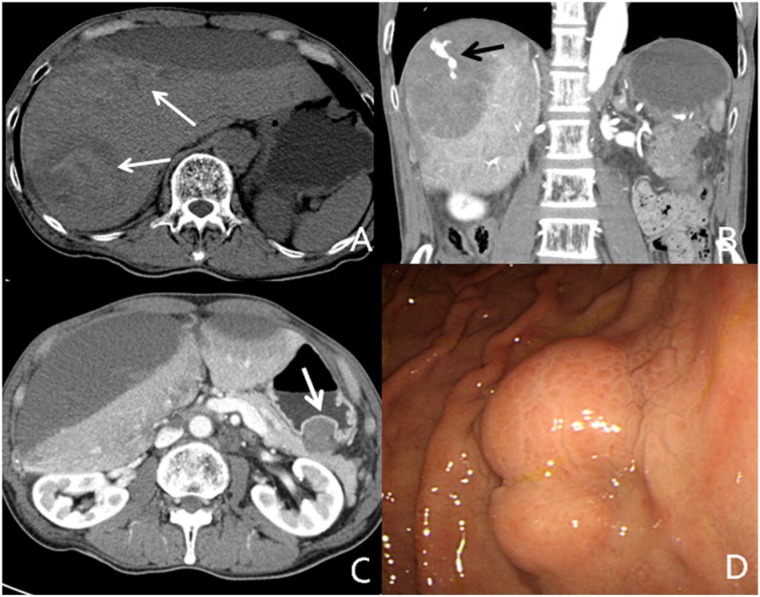
A 65-year-old man with a surgically diagnosed rupture of a hepatic metastasis from a gastric GIST. (A) An axial unenhanced CT section shows a large subcapsular hematoma with high-density sentinel clots (arrow) in the right posterior hepatic lobe. (B) Contrast-enhanced CT with coronal reconstruction shows a spherical heterogeneous low-density hepatic mass with active contrast leakage (arrow) adjacent to the hematoma, which was diagnosed as a ruptured hepatic metastasis after resection. (C) An abnormal low-density gastric mass is shown in the posterior body with normal overlying gastric mucosa (arrow). (D) A dumbbell-shaped submucosal mass is revealed by gastrofiberscopy at the level of the gastric body.

Abdominal multidetector computed tomography (CT) showed a large hematoma in the intrahepatic and subcapsular regions of the liver. A spherical hepatic mass identified in the posterior segment of the liver was connected to the hematoma (Figure 1). The mass was a poorly enhancing, relatively well-encapsulated hepatic tumor. However, capsular disruption, indicated by active contrast leakage, was noted on the posterosuperior wall. An incidentally detected gastric mass in the posterior body measuring approximately 2.7 × 2.6 cm had prominent enhancement of the normal overlying gastric mucosa, suggesting that the tumor originated from the gastric subepithelium (Figure 1).

**Figure 2.**
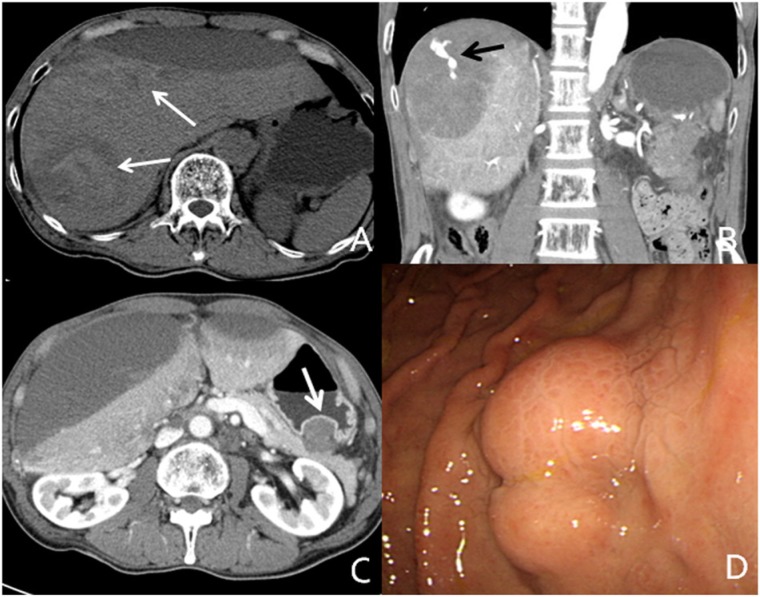
On emergency angiography, hepatic tumoral vessels are shown arising from the anterior segmental branches of the right hepatic artery with a pear-shaped region of active contrast leakage (arrow).

An emergency angiography showed hepatic tumor vessels emerging from the anterior segmental branches of the right hepatic artery with abnormal pear-shaped active contrast leakage (Figure 2). Diffuse hypervascular tumor staining was present in the right hepatic dome. After superselection of the right hepatic artery with a microcatheter and microguidewire, a mixture of 3 cm^3^ Lipiodol and 20 mg Doxorubicin (Adriamycin, Ildong, South Korea) was slowly infused. Because most of the Lipiodol mixture leaked, complete occlusion of the feeder vessel at the leakage site was performed using a 50% mixture of *n*-butyl cyanoacrylate (Histoacryl, B-Braun, Melsungen, Germany).

Postembolization celiac angiography showed no further visible contrast leakage, exhibiting only residual tumor staining. The vital signs of the patient were stabilized, and an abnormal dumbbell-shaped gastric submucosal mass was identified during gastrofiberscopy at the level of the gastric body (Figure 1D). Three days after embolization, the vital signs of the patient destabilized. A large amount of hemoperitoneum was identified by CT and was suspected to be a ruptured subcapsular hematoma. A posterior sectionectomy of the liver and a wedge resection of the gastric tumor were performed during emergency surgery. Histology showed that the gastric and hepatic tumors were composed of short spindle-shaped cells with abundant eosinophilic cytoplasm and cigar-shaped nuclei.

The cellularity was relatively moderate, and the frequency of mitotic figures was 15 mitoses per 50 high-power fields. In immunohistochemical studies, the tumors were diffusely immunoreactive for CD 34 and C-kit (CD 117) and negative for SMA, Desmin, and S-100 protein. Based on these pathologic and immunohistochemical findings, a diagnosis of ruptured hepatic metastasis from a malignant gastric GIST was made. The patient had an uneventful postoperative course over the subsequent 6-month period.

## Discussion

Hemoperitoneum secondary to spontaneous ruptured hepatic metastases from primary tumors of the lung, pancreas, stomach, kidney, breast, prostate, testicle, gallbladder, skin (melanoma), and nasopharynx as well as choriocarcinoma and hepatic lymphoma has been reported.^1^

GISTs are common mesenchymal tumors arising from the gastrointestinal tract with an incidence of 10 to 20 cases per million.^2^ Most individuals are older than 50 years at the time of presentation. GISTs rarely occur in patients younger than 40 years of age.^3^ GISTs are most common in the stomach (70% of cases), followed by the small bowel (20% to 30%), anorectum (7%), colon, esophagus, omentum, and mesentery.^2^ Based on several immunohistochemical studies, it has become widely accepted that GISTs differentiate from the interstitial cells of Cajal.^4^

Immunohistochemical analysis of the tumor in our case revealed diffusely positive immunoreactivity for C-kit (CD 117), tyrosine kinase growth factor receptor, and CD 34 but negative immunoreactivity for smooth muscle actin, Desmin, and S-100 protein. These results indicate that the tumor had differential features of the interstitial cells of Cajal, leiomyomas, leiomyosarcomas, schwannomas, and neurofibromas.^5^ The clinical features of GISTs over long follow-up periods have been investigated. Most GISTs (62.6%) were identified incidentally through endoscopic screening. The most common symptom was pain, followed by gastrointestinal bleeding, signs of obstruction, and masses.

Malignant GISTs commonly metastasize to the liver or peritoneum. In contrast, lymph node and extra-abdominal metastases are rare. The liver is the most common metastatic site at both initial presentation and relapse of the disease.^6^ However, the rupture of a hematoma and the development of hemoperitoneum from a metastatic GIST are very rare. These symptoms are frequently associated with primary hepatic tumors, such as hepatocellular carcinoma or hepatic adenoma, which have greater vascularity than metastatic lesions. Since the first reported case of a ruptured hepatic metastasis from GIST by Suzuki et al in 2003, only 2 additional cases (one in the greater omentum and one in the jejunum)^7,8^ have been documented in the English literature. At imaging, the diagnosis of a hemorrhagic metastasis is suggested if blood is identified in one or more liver lesions in a patient with known hepatic metastases or a known primary hepatic tumor. If the hemorrhage is severe, a subcapsular hematoma or hemoperitoneum may also be noted. The presence and extent of intrahepatic or subcapsular hematoma and hemoperitoneum can be easily identified using CT and may appear as a hyperattenuating mass or a mass with high-density material or hyperdense ascites. During the first 24 to 72 hours, acute hematomas are hyperattenuating on precontrast CT scans. Afterward, the attenuation decreases, and a pseudocapsule may develop after the subacute stage.

The treatment of hemoperitoneum depends on the size of the hepatic tumors, their location, and the rate of bleeding. Controlling the hemorrhage is the main objective. Regardless of the treatment, the prognosis is very poor. One patient died 3 months after the recurrence of bleeding, and another patient survived for less than 6 weeks.^9^

The precise cause of the spontaneous rupture of a GIST or metastatic GIST is unknown. The rupture may originate at a wall of a mass that is weakened by cystic or hemorrhagic degeneration. It is generally believed that the malignant potential of a GIST, such as that of other mesenchymal tumors, is related to hypercellularity, nuclear atypia, and mitotic activity. Local and/or distant metastases can develop from a potentially malignant GIST. However, it is often difficult to establish a preoperative diagnosis. In most cases, the clinical behavior of a GIST can be predicted with relative accuracy using a combination of tumor size and mitotic activity. Gastric GISTs measuring at least 10 cm or with more than 5 mitoses per 50 high-power fields have high risk for progression, metastatic spread, or relapse after resection. All GISTs are considered potentially malignant. Once GISTs recur, metastasize, or invade adjacent organs, they are considered malignant.

Complete surgical excision with a wide margin of normal tissue is the treatment of choice for GISTs. Regional node resection is not always required because nodal metastases are not usually associated with GISTs. If a patient with a GIST has metastatic lesions in the peritoneal cavity and/or the liver, complete resection of the lesions may improve survival. Imatinib (Imatinib mesylate [Gleevec]; Novartis Pharma, Basel, Switzerland) is a new molecularly targeted tyrosine kinase receptor blocker that targets tumor cells specifically, limits their growth, induces apoptosis, and leads to a dramatic treatment response and markedly improved long-term survival of patients with GISTs.^10^

In conclusion, we reported a rare case of a spontaneously ruptured hepatic metastasis from a malignant gastric GIST that presented as hemoperitoneum. We also described the proper treatment of the condition.
